# SATB2 and MDM2 Immunoexpression and Diagnostic Role in Primary Osteosarcomas of the Jaw

**DOI:** 10.3390/dj10010004

**Published:** 2021-12-30

**Authors:** Adepitan A. Owosho, Adeola M. Ladeji, Olufunlola M. Adesina, Kehinde E. Adebiyi, Mofoluwaso A. Olajide, Toluwaniyin Okunade, Jacob Palmer, Temitope Kehinde, Jeffrey A. Vos, Grayson Cole, Kurt F. Summersgill

**Affiliations:** 1 Missouri School of Dentistry and Oral Health, A.T. Still University, Kirksville, MO 63501, USA; jacobpalmer@atsu.edu; 2 Department of Oral Pathology and Oral Medicine, Faculty of Dentistry, Lagos State University, Lagos 101233, Nigeria; adeola.ladeji@gmail.com (A.M.L.); kenad93@gmail.com (K.E.A.); foluabim@gmail.com (M.A.O.); 3 Department of Oral Medicine and Oral Pathology, Faculty of Dentistry, Obafemi Awolowo University, Ile-Ife 220282, Nigeria; tunrayoade@yahoo.com (O.M.A.); toluwaniokunade@gmail.com (T.O.); 4 Department of Pathology, Anatomy and Laboratory Medicine, West Virginia University, Morgantown, WV 26506, USA; temitope.kehinde@hsc.wvu.edu (T.K.); jvos@hsc.wvu.edu (J.A.V.); 5 Department of Diagnostic Sciences, School of Dental Medicine, University of Pittsburgh, Pittsburgh, PA 15213, USA; colegg@upmc.edu (G.C.); kfs8@pitt.edu (K.F.S.)

**Keywords:** ossifying fibroma, fibrous dysplasia, *MDM2* amplification, jaw tumors, immunohistochemistry

## Abstract

Primary osteosarcomas of the jaw (OSJ) are rare, accounting for 6% of all osteosarcomas. This study aims to determine the value of SATB2 and MDM2 immunohistochemistry (IHC) in differentiating OSJ from other jawbone mimickers, such as benign fibro-osseous lesions (BFOLs) of the jaw or Ewing sarcoma of the jaw. Certain subsets of osteosarcoma harbor a supernumerary ring and/or giant marker chromosomes with amplification of the 12q13–15 region, including the murine double-minute type 2 (MDM2) and cyclin-dependent kinase 4 (CDK4) genes. Special AT-rich sequence-binding protein 2 (SATB2) is an immunophenotypic marker for osteoblastic differentiation. Cases of OSJ, BFOLs (ossifying fibroma and fibrous dysplasia) of the jaw, and Ewing sarcoma of the jaw were retrieved from the Departments of Oral Pathology and Oral Medicine, Faculty of Dentistry, Obafemi Awolowo University and Lagos State University College of Medicine, Nigeria. All OSJ retrieved showed histologic features of high-grade osteosarcoma. IHC for SATB2 (clone EP281) and MDM2 (clone IF2), as well as fluorescence in situ hybridization (FISH) for MDM2 amplification, were performed on all cases. SATB2 was expressed in a strong intensity and diffuse staining pattern in all cases (11 OSJ, including a small-cell variant, 7 ossifying fibromas, and 5 fibrous dysplasias) except in Ewing sarcoma, where it was negative in neoplastic cells. MDM2 was expressed in a weak to moderate intensity and scattered focal to limited diffuse staining pattern in 27% (3/11) of cases of OSJ and negative in all BFOLs and the Ewing sarcoma. *MDM2* amplification was negative by FISH in interpretable cases. In conclusion, the three cases of high-grade OSJs that expressed MDM2 may have undergone transformation from a low-grade osteosarcoma (LGOS). SATB2 is not a dependable diagnostic marker to differentiate OSJ from BFOLs of the jaw; however, it could serve as a valuable diagnostic marker in differentiating the small-cell variant of OSJ from Ewing sarcoma of the jaw, while MDM2 may be a useful diagnostic marker in differentiating OSJ from BFOLs of the jaw, especially in the case of an LGOS or high-grade transformed osteosarcoma.

## 1. Introduction

Osteosarcoma is the most common malignant primary bone tumor arising from mesenchymal osteoblasts that produce neoplastic osteoid or immature bone [1]. It commonly affects children and adolescents, typically involving metaphyses of long bones, such as the femur, tibia, and humerus. The predisposing conditions to osteosarcoma are Li-Fraumeni syndrome, congenital poikiloderma, prior radiation to bone, and bone diseases such as Paget’s disease of bone, chronic osteomyelitis, bone infarct, and fibrous dysplasia [2,3,4,5,6,7]. Based on its growth pattern, osteosarcoma can be further identified as being either an intramedullary or surface (parosteal “low-grade”, periosteal “intermediate-grade”, and high-grade) subtype. With regard to histomorphology, osteosarcoma can be classified as osteoblastic, chondroblastic, or fibroblastic. It can also be classified as other, rarer forms, such as the small-cell variant, epithelioid, giant cell-rich, telangiectatic, and well-differentiated (low-grade intraosseous). Despite advances in therapy, the survival rate of osteosarcoma patients remains dismal, particularly for patients that develop distant metastasis [5,8,9].

Primary osteosarcomas of the jaw (OSJ) are rare, accounting for 6% of all osteosarcomas [1]. OSJ occurs in individuals who are older by one to two decades than the average age of onset in extragnathic sites [1,10]. The mandible is the most common location for OSJ, and the chondroblastic form is the most common histomorphologic type of OSJ. OSJ has a better prognosis compared to osteosarcomas of extragnathic sites, due to a less frequent occurrence of distant metastasis [10,11].

Special AT-rich sequence-binding protein 2 (SATB2) is a DNA-binding protein that functions as a nuclear matrix-associated transcription factor and is involved in transcriptional regulation and chromatin remodeling. SATB2 is expressed in glandular cells lining the lower gastrointestinal tract (colon and rectum), subsets of lymphoid cells, subsets of neuronal cells in the cerebral cortex and hippocampus, cells lining the epididymis, and cells lining the seminiferous ducts of the testis [12]. Disruption/de novo mutation of the *SATB2* gene has been etiologically implicated in both cleft palate and Pierre Robin syndrome [13,14,15]. This disruption in *SATB2* has shown significant dysregulation in skeletogenesis in mice models, due to the downregulation of downstream “osteoblast master regulator” target genes, such as *RUNX2* and *ATF4* [16,17]. SATB2 is a sensitive immunophenotypic marker for tumors of osteoblastic differentiation and colorectal carcinomas [18,19]. The SATB2 immunohistochemical marker has been shown to help distinguish osteosarcomas of long bones, including the small-cell variant, from their malignant bone tumor mimickers, such as Ewing sarcomas [20].

Studies have documented that low-grade (parosteal and well-differentiated) osteosarcomas harbor a supernumerary ring and/or giant marker chromosomes with amplification of the 12q13–15 region, including the murine double-minute type 2 (*MDM2*) and cyclin-dependent kinase 4 (*CDK4*) genes, as well as subsequent overexpression of their respective proteins [21]. Low-grade osteosarcomas can transform into high-grade osteosarcomas and still preserve the amplification of chromosome 12q and overexpression of *MDM2* and *CDK4* [21]. MDM2 and CDK4 immunohistochemistry has been shown to be a valuable tool in differentiating low-grade osteosarcomas from other primary fibro-osseous lesions of long bones [22]. *MDM2* is a gene encoded on the chromosome 12q13–14 region. *MDM2* is a master regulator of p53 and contributes to tumorigenesis by inhibiting the activity of this tumor suppressor through the following mechanisms: blocking the transcription of p53 by a 483-amino acid protein it encodes for by binding to the N-terminal transcription region of the tumor suppressor, accelerating the degradation of the p53 protein, and directly driving p53 from the nucleus [23,24,25,26]. *MDM2* amplification has been well documented in well-differentiated liposarcomas/atypical lipomatous tumors, intimal sarcomas, and hematologic malignancies [27,28,29].

The aims of this study are to report the clinicopathologic features of OSJ and to determine the value of SATB2 and MDM2 immunohistochemistry in differentiating OSJ from other common jawbone mimickers. We investigated these in OSJ (including a small-cell variant), benign fibro-osseous lesions (fibrous dysplasia and ossifying fibroma) of the jaw, and a primary Ewing sarcoma of the jaw.

## 2. Materials and Methods

The pathology files of the Department of Oral Medicine and Oral Pathology, Faculty of Dentistry, Obafemi Awolowo University, Ile-Ife, Nigeria and the Department of Oral Pathology and Oral Medicine, Faculty of Dentistry, Lagos State University College of Medicine, Lagos, Nigeria were searched for histologically-diagnosed primary osteosarcomas of the jaw from 2016 to 2019. The archived formalin-fixed, paraffin-embedded osteosarcomas were retrieved, and the hematoxylin and eosin slides were reviewed and classified based on their histomorphology. The following clinical information was retrieved: age at diagnosis, gender, jawbone involved (mandible or maxilla), and the duration of time between the onset of the first symptom of the tumor and its ensuing pathologic diagnosis. Cases of benign fibro-osseous lesions (fibrous dysplasia and ossifying fibroma) of the jaw and a primary Ewing sarcoma of the jaw were retrieved from the former institution.

Immunostaining for SATB2 and MDM2 was performed on 4-µm-thick, paraffin-embedded sections of osteosarcomas, benign fibro-osseous lesions (fibrous dysplasia and ossifying fibroma) of the jaw, and a primary Ewing sarcoma of the jaw at the following institutions: University of Pittsburgh Medical Center, Pittsburgh, PA, USA and West Virginia University Health Sciences Center, Morgantown, WV, USA, respectively. The following antibodies used were: MDM2 (mouse monoclonal antibody, clone IF2, dilution 1:25, Cell Marque, Rocklin, CA, USA) and SATB2 (rabbit monoclonal primary antibody, clone EP281, dilution 1:25, Cell Marque, Rocklin, CA, USA). Only nuclear staining was considered positive in the interpretation of SATB2 and MDM2. Distribution of staining was scored as: 0 to less than 1% (negative), greater than 1 to 10% (focal), greater than 10 to 100% (diffuse), and scoring 0, no staining; 1, <33%; 2, 34–66%; 3, 67–100%, and intensity was semiquantitatively graded as weak, moderate, or strong for both SATB2 and MDM2. Staining of any intensity of at least 1% of neoplastic cells was considered positive. The percentage of positive tumor cell nuclei was approximated by visually scanning the slides at medium power. Fluorescence in-situ hybridization was performed using dual-color, locus-specific identifier (LSI) probes for *MDM2* (Abbott Molecular Inc., Des Plains, IL, USA) on all cases at the University of Pittsburgh Medical Center, Pittsburgh, PA, USA. This study was exempted from review by the institutional review board of A.T. Still University, Kirksville, MO, USA.

## 3. Results

### 3.1. Clinicopathologic Findings

A total of 11 cases of primary osteosarcoma of the jaw were retrieved. The patients’ clinicopathologic characteristics are presented in Table 1. The female-to-male ratio was 1.2:1. The average age of patients at diagnosis was 29 years (range, 10–49 years). Seven cases involved the mandible and four cases involved the maxilla. The histologic subtypes were: chondroblastic (*n* = 5), osteoblastic (*n* = 3), fibroblastic (*n* = 2), and small cell (*n* = 1). All OSJ retrieved showed histologic features of intermediate to high-grade OS. The 10 tumors with reported sizes ranged from 5–16 cm (mean, 9.1 cm). The duration of time between the onset of the first symptom of each tumor and its ensuing pathologic diagnosis ranged from two weeks to two years (mean, 5.35 months). Twelve benign fibro-osseous lesions (seven ossifying fibromas and five fibrous dysplasias) of the jaw and a primary Ewing sarcoma of the jaw were also retrieved and evaluated for SATB2 and MDM2 immunohistochemistry (IHC).

### 3.2. SATB2 Status

**Figure 1:**
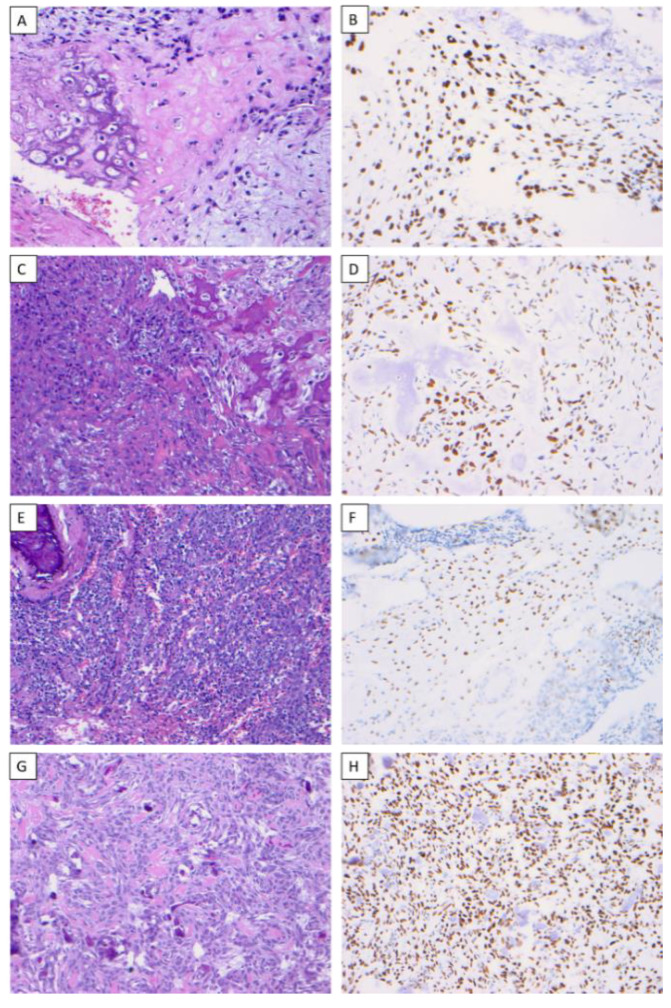
SATB2 expression in osteosarcoma. Chondroblastic osteosarcoma of the mandible (H & E) (×200) (**A**), chondroblastic osteosarcoma demonstrating nuclear immunoreactivity to SATB2 in a diffuse staining pattern with strong intensity (×200) (**B**), osteoblastic osteosarcoma of the maxilla (H & E) (×200) (**C**), osteoblastic osteosarcoma demonstrating nuclear immunoreactivity to SATB2 in a diffuse staining pattern with strong intensity (×200) (**D**), small-cell osteosarcoma of the mandible (H & E) (×200) (**E**), small-cell osteosarcoma demonstrating nuclear immunoreactivity to SATB2 in a diffuse staining pattern with strong intensity (×200) (**F**), fibroblastic osteosarcoma of the maxilla (H & E) (×200) (**G**), fibroblastic osteosarcoma demonstrating nuclear immunoreactivity to SATB2 in a diffuse staining pattern with strong intensity (×200) (**H**).

**Figure 2:**
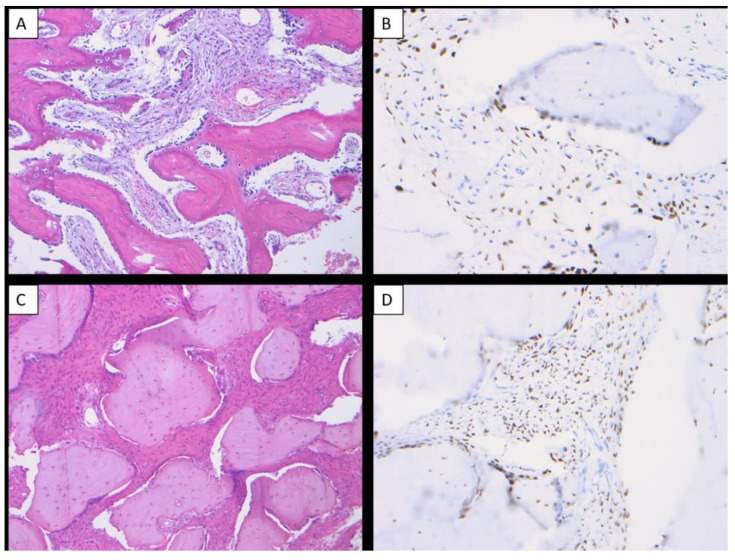
Benign fibro-osseous lesions of the jaw were immunoreactive to SATB2. Ossifying fibroma of the jaw (H & E ×100) (**A**), ossifying fibroma demonstrating nuclear immunoreactivity with strong intensity and diffuse staining pattern to SATB2 in the stromal plump and spindle cells (×200) (**B**), fibrous dysplasia of the jaw (H & E ×100) (**C**), fibrous dysplasia demonstrating nuclear immunoreactivity with strong intensity and diffuse staining pattern to SATB2 in the stromal plump and spindle cells (×200) (**D**).

**Figure 3:**
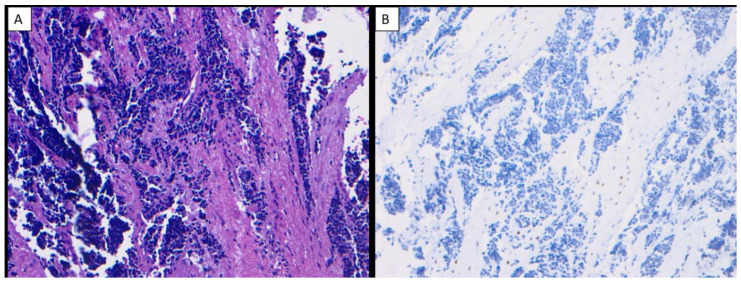
Ewing sarcoma of the jaw (H & E ×200) (**A**), Ewing sarcoma of the jaw showed no nuclear immunoreactivity to SATB2 in any neoplastic cells (×200) (**B**).

All cases of OSJ (*n* = 11) demonstrated nuclear immunoreactivity with strong intensity and a diffuse staining pattern to SATB2 in the stromal plump and spindle cells (Figure 1A–H). All cases of benign fibro-osseous lesions of the jaw (ossifying fibroma (*n* = 7) and fibrous dysplasia (*n* = 5)) also demonstrated nuclear immunoreactivity with a strong intensity and diffuse staining pattern to SATB2 in the stromal plump and spindle cells (Figure 2A–D). The case of primary Ewing sarcoma of the jaw showed no nuclear immunoreactivity in any neoplastic cells (Figure 3A,B). The staining distribution results are presented in Table 2.

### 3.3. MDM2 Status

**Figure 4:**
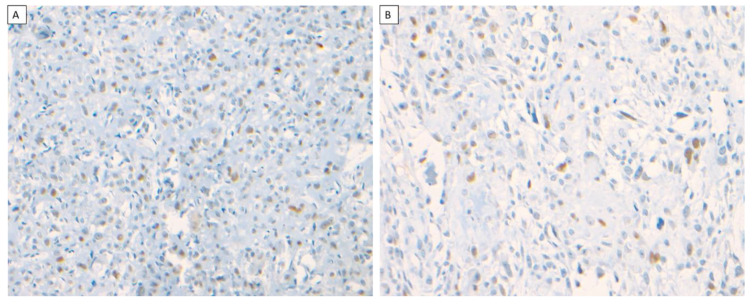
MDM2 expression in osteosarcoma. Osteosarcoma of the mandible in a 49-year-old male patient with nuclear expression of MDM2 in a diffuse staining pattern with moderate intensity (×200) (**A**), osteosarcoma of the mandible in a 19-year-old female patient with nuclear expression of MDM2 in a diffuse staining pattern with moderate intensity (×200) (**B**).

MDM2 IHC was only positive in 27% (3/11) of the osteosarcoma cases (Cases 1–3). All three cases occurred in the mandible. Cases 1 and 2 demonstrated nuclear immunoreactivity with a weak-to-moderate intensity and limited diffuse staining pattern to MDM2 in the stromal plump and spindle cells (Figure 4A,B), while Case 3 demonstrated nuclear immunoreactivity with a moderate intensity and scattered focal staining pattern to MDM2 in the stromal plump and spindle cells. All cases of benign fibro-osseous lesions of the jaw showed no nuclear immunoreactivity in the stromal cells or bony cells, and the case of primary Ewing sarcoma of the jaw showed no nuclear immunoreactivity in any neoplastic cells. The staining distribution results are presented in Table 2.

*MDM2* FISH analysis was feasible with an interpretable signal in 5 of the 24 investigated cases: 2 of 11 osteosarcoma cases, 2 of 7 ossifying fibroma cases, and 1 of 5 fibrous dysplasia cases. Gene amplification of *MDM2* was not detected in any of the five interpretable tumors analyzed. Notably, in the nine non-informative, FISH-analyzed osteosarcoma cases, there were three osteosarcoma cases with MDM2 immunohistochemical expression as described above.

## 4. Discussion

Osteosarcoma is the most common malignant primary bone tumor, typically involving metaphyses of long bones, such as the femur, tibia, and humerus [1]. Primary osteosarcomas of the jaw (OSJ) are rare, accounting for 6% of all osteosarcomas [1]. Low-grade osteosarcomas (LGOS) are characterized by plump/spindle cell stroma with low-to-moderate cellularity and well-differentiated anastomosing bone trabeculae, which can be easily confused with benign fibro-osseous lesions (BFOLs), such as ossifying fibroma and fibrous dysplasia, in the setting of a limited/small biopsy.

In this study, we evaluated the value of SATB2 and MDM2 IHC in differentiating OSJ from other common jawbone mimickers, such as benign fibro-osseous lesions (fibrous dysplasia and ossifying fibroma) of the jaw and primary Ewing sarcoma of the jaw. This is the first study evaluating both SATB2 and MDM2 expression in OS, BFOLs, and Ewing sarcoma of the jaw. SATB2 was positive in all 11 OSJs, including the small-cell variant, and all 12 BFOLs of the jaw evaluated, but in the case of Ewing sarcoma of the jaw, it was negative in neoplastic cells. Our findings are corroborated by similar studies. In a recent study by Grad-Akrish et al., it was shown that SATB2 was positive in all 15 OSJs and all 42 BFOLs of the jaw [30]. Another study by Connor and Hornick reported SATB2 positivity in all extragnathic osteosarcomas, fibrous dysplasias, and benign bone-forming tumors, such as osteoblastomas and osteoid osteomas [31]. Also, Machado et al., in their report, revealed that 90.4% of OSs were positive for SATB2 and 98.7% of 371 genetically confirmed Ewing sarcomas were negative for SATB2 [20]. Our study found that 27% (3/11) of high-grade OSJs were positive for MDM2, while all BFOLs of the jaw and a Ewing sarcoma of the jaw were negative for MDM2. Other studies share similar results: a recent study by Lott Limbach et al. showed that MDM2 was expressed in 63% (7/11) of craniofacial OSs and not expressed in any BFOLs of the jaw [32]. Meanwhile, Guerin et al. showed that MDM2 was expressed in 8% (3/36) of craniofacial OSs, but they found no expression of MDM2 in any of the benign bone-forming tumors [21]. A study by Lopes et al. revealed MDM2 expression in 89% (8/9) of OSJs [33]. Junior et al. found that MDM2 was expressed in 24% (6/25) of OSJs [34] (Table 3). Additionally, Dujardin et al. reported no expression of MDM2 in any BFOLs of the jaw evaluated [22]. Notably, Tabareau-Delalande et al. identified *MDM2* amplification by qPCR in 26% (12/47) of craniofacial BFOLs, and interestingly, 75% (9) of the amplified cases were juvenile ossifying fibromas; however, MDM2 expression was not observed in any of the 47 craniofacial BFOLs even though all the controls (15 well-differentiated/dedifferentiated liposarcomas) showed expression of MDM2 [35]. The wide disparity in the percentage of OSJs expressing MDM2 among the different studies could be due to differences in tissue fixation, decalcification, antibody cloning, antibody dilution, and antigen retrieval. For instance, Lott Limbach et al. utilized two different clones (IF2 and SMP14) of the MDM2 antibody for the same cases, and the IF2 clone was expressed in 36% (4/11) of the cases, while the SMP14 clone was expressed in 86% (6/7) of craniofacial OSs [32].

The presence of *MDM2* amplification in OS presents a therapeutic opportunity. MDM2 inhibitors, such as Nutlin-3a, suppress proliferation and promote apoptosis in OS and other cancers [36,37]. This small-molecule MDM2 inhibitor disrupts the p53-MDM2 interaction by acting as a high-affinity compound-antagonist to MDM2 [37], but the impact of MDM2 inhibitors as single-agent therapeutics has limited antineoplastic activity in clinical trials. Yet, if combined with other therapeutic agents, its antineoplastic activity could be considerably intensified [38,39,40,41].

The limitation of this study is that we did not evaluate CDK4, which may have given us the true representation of amplification of the 12q13–15 region in the OSJs in our study. Several studies have demonstrated that, when it comes to amplification of the 12q13–15 region, the overexpression of MDM2 and CDK4 proteins is mutually exclusive [22,32,42]. In conclusion, the three cases of high-grade OSJ that expressed MDM2 may have undergone transformation from LGOS. SATB2 is not a reliable diagnostic marker to differentiate OSJ from BFOLs of the jaw; however, it could serve as a valuable diagnostic marker in differentiating the small-cell variant of OSJ from Ewing sarcoma of the jaw, while MDM2 may be a useful diagnostic marker in differentiating OSJ from BFOLs of the jaw, especially in the setting of an LGOS or high-grade transformed OS. Lastly, the absence of MDM2, immunostaining does not necessarily signify that a tumor is not an OS.

## Figures and Tables

**Table 1:** Clinicopathologic features of osteosarcomas of the jaw.

Case No.	Age	Sex	Location	Size (cm)	SATB2 IHC	MDM2 IHC	*MDM2* FISH
1	49	M	Mandible	5	Positive	Positive	Failed
2	19	F	Mandible	10	Positive	Positive	Failed
3	16	M	Mandible	NA	Positive	Positive, focal	Failed
4	35	F	Mandible	9.5	Positive	Negative	Failed
5	35	M	Maxilla	10	Positive	Negative	Failed
6	23	F	Maxilla	5	Positive	Negative	Failed
7	10	M	Mandible	6.5	Positive	Negative	Failed
8	42	F	Maxilla	12	Positive	Negative	Failed
9	17	F	Mandible	16	Positive	Negative	Failed
10	42	F	Maxilla	5	Positive	Negative	No amplification
11	31	M	Mandible	12	Positive	Negative	No amplification

NA—not available.

**Table 2:** Comparative SATB2 and MDM2 immunohistochemistry in primary osteosarcomas, Ewing sarcoma, and benign fibro-osseous lesions of the jaw.

Diagnosis	No. of Cases	No. (%) of Cases Positive for SATB2				No. (%) of Cases Positive for MDM2			
		Score 0	Score 1	Score 2	Score 3	Score 0	Score 1	Score 2	Score 3
Chondroblastic OSJ	5	0	1 (20)	2 (40)	2 (40)	4 (80)	1 (20)	0	0
Osteoblastic OSJ	3	0	0	0	3 (100)	2 (67)	1 (33)	0	0
Fibroblastic OSJ	2	0	0	1 (50)	1 (50)	1 (50)	1 (50)	0	0
Small-cell OSJ	1	0	0	1 (100)	0	1 (100)	0	0	0
Ewing sarcoma	1	1 (100)	0	0	0	1 (100)	0	0	0
Ossifying fibroma	7	0	3 (43)	2 (28)	2 (28)	7 (100)	0	0	0
Fibrous dysplasia	5	0	4 (80)	1 (20)	0	5 (100)	0	0	0

OSJ—primary osteosarcoma of the jaw.

**Table 3:** Literature review of SATB2 and MDM2 in craniofacial and jaw osteosarcoma.

Articles	SATB2 IHC	MDM2 IHC
Grad-Akrish et al. [30]	15/15 (100%) all moderate or strong diffuse	NP
Lott Limbach et al. [32]	NP	7/11 (63%; all weak focal)
Guerin et al. [21]	NP	3/36 (8%)
Lopes et al. [33]	NP	8/9 (89%)
Junior et al. [34]	NP	6/25 (24%)
Owosho et al. (current)	11/11 (100%) all strong diffuse	3/11 (27%; two moderate diffuse, one weak focal)

IHC—immunohistochemistry, NP—not performed.
